# Artificial Neural Networks to Predict Metabolic Syndrome without Invasive Methods in Adolescents

**DOI:** 10.3390/jcm13195914

**Published:** 2024-10-03

**Authors:** Antonio Costa Júnior, Ana Karina França, Elisângela dos Santos, Victor Silveira, Alcione dos Santos

**Affiliations:** 1 Coordenação do Curso de Medicina, Centro de Ciências de Pinheiro, Universidade Federal do Maranhão, São Luís 65200-000, Brazil; 2 Programa de Pós-Graduação em Saúde Coletiva, Departamento de Saúde Pública, Universidade Federal do Maranhão, São Luís 65020-070, Brazil; ana.franca@ufma.br (A.K.F.); victor.ncs@discente.ufma.br (V.S.); alcione.miranda@ufma.br (A.d.S.); 3 Departamento de Enfermagem, Universidade Federal do Maranhão, São Luís 65080-805, Brazil; milhomem.elisangela@ufma.br

**Keywords:** metabolic syndrome, adolescents, screening, primary health care, artificial intelligence

## Abstract

**Background/Objectives:** The prevalence of metabolic syndrome (MetS) is increasing worldwide, and an increasing number of cases are diagnosed in younger age groups. This study aimed to propose predictive models based on demographic, anthropometric, and non-invasive clinical variables to predict MetS in adolescents. **Methods**: A total of 2064 adolescents aged 18–19 from São Luís-Maranhão, Brazil were enrolled. Demographic, anthropometric, and clinical variables were considered, and three criteria for diagnosing MetS were employed: Cook et al., De Ferranti et al. and the International Diabetes Federation (IDF). A feed-forward artificial neural network (ANN) was trained to predict MetS. Accuracy, sensitivity, and specificity were calculated to assess the ANN’s performance. The ROC curve was constructed, and the area under the curve was analyzed to assess the discriminatory power of the networks. **Results**: The prevalence of MetS in adolescents ranged from 5.7% to 12.3%. The ANN that used the Cook et al. criterion performed best in predicting MetS. ANN 5, which included age, sex, waist circumference, weight, and systolic and diastolic blood pressure, showed the best performance and discriminatory power (sensitivity, 89.8%; accuracy, 86.8%). ANN 3 considered the same variables, except for weight, and exhibited good sensitivity (89.0%) and accuracy (87.0%). **Conclusions**: Using non-invasive measures allows for predicting MetS in adolescents, thereby guiding the flow of care in primary healthcare and optimizing the management of public resources.

## 1. Introduction

The prevalence of metabolic syndrome (MetS) is increasing in younger age groups, with adverse future outcomes [1,2,3] including increased morbidity and mortality associated with cardiovascular disease [2,4].

MetS is defined as the presence of three or more of the following components: elevated triglycerides, blood pressure, and fasting glucose, reduced high-density lipoprotein (HDL) cholesterol, and central fat deposition represented by waist circumference (WC), which may be a mandatory component for MetS, depending on the criteria adopted [5,6].

Researchers and societies use different criteria to define MetS [1,3]. Nevertheless, despite evaluating the same components, the criteria differ in two key aspects: the cut-off points used for each component, and an abnormal WC being mandatory for a diagnosis of MetS [3,7].

Consequently, the estimated prevalence of MetS may vary within the same population, depending on the diagnostic criteria adopted [8]. Vanlanker et al. [9] observed a variation ranging between 2.7 and 3.8% among European adolescents aged 12.5 to 17.0 years.

Early diagnosis of MetS and its risk factors is essential for more effective interventions that lead to favorable outcomes [3,10]. However, some biochemical tests are required regardless of the criteria to diagnose MetS [5,6]. Although these are not particularly complex, they are invasive, require patients to return for medical appointments, and are costly to the public healthcare system.

In primary healthcare, applying Artificial Intelligence has the potential to screen patients likely to have MetS, based on non-invasive measurements [4]. The construction of predictive models, using artificial neural networks (ANN) based on metabolic abnormalities that define MetS and other anthropometric measurements, has demonstrated satisfactory performance in predicting MetS. These models can be used as supplementary tools to identify cardiometabolic risk [11,12]. However, the predictive models developed to date are for Asian adults [11] and are based on a single defining criterion for MetS [4,10,11]. Moreover, these predictive models include invasive and costly variables, which may delay diagnosis and treatment.

Thus, this study aims to predict MetS in adolescents using ANNs based on non-invasive demographic, clinical, and anthropometric variables.

## 2. Materials and Methods

This is a cross-sectional study with a dataset from the 1997–1998 birth cohort in São Luís-Maranhão, Brazil. Further details on the methodological approaches of this cohort can be found in Confortin et al. [13].

The sample consisted of 2515 adolescents assessed at 18–19 years old, of whom 687 were from the original cohort and 1828 from an open cohort composed of people born in São Luís-MA in 1997. The purpose of including new participants was to increase the sample size. They were randomly selected from the Live Birth Information System (Sistema de Informações de Nascidos Vivos), including children born in 1997, and identified at schools and universities and through social networks. A total of 451 participants were excluded due to incomplete information or inconsistencies in the variables of interest. The final sample included 2064 adolescents aged 18–19 from São Luís-Maranhão, Brazil.

The following variables were used in this study: age (years), sex (male or female), systolic blood pressure (SBP) (mmHg), diastolic blood pressure (DBP) (mmHg), weight (kg), height (cm), WC (cm), serum glucose (mmol/L), triglycerides (mmol/L), and HDL cholesterol (mmol/L).

For SBP and DBP, the mean value of three measurements was considered, taken after five minutes of rest, using the Omron HEM742INT device (Omron^®^, São Paulo, Brazil). Glycemia and serum levels of HDL cholesterol and triglycerides were measured using the automated enzymatic colorimetric method using the Cobas c501 device (Roche^®^, São Paulo, Brazil). Weight was measured using a high-precision scale integrated with BOD POD Gold Standard equipment (COSMED Metabolic Company^®^, Rome, Italy). Height was measured using a portable stadiometer (AlturaExata^®^, Belo Horizonte, Brazil). WC was obtained from a three-dimensional image of the body using a 3-Dimensional Photonic Scanner ([TC] Labs^®^, Cary, NC, USA). Body mass index (BMI) was calculated as follows: body weight (kg) divided by height squared (m^2^).

To identify MetS in participants, the three most widely used specific criteria for adolescents were considered: 1. Cook et al. [14]; 2. De Ferranti et al. [15]; and 3. International Diabetes Federation (IDF) [16]. These classifications considered elevated triglycerides, fasting glucose, SBP and/or DBP, WC, and low HDL cholesterol levels. The IDF criteria for identifying MetS require the presence of central obesity, as measured by WC, in addition to at least two metabolic abnormalities. The criteria established by Cook et al. and De Ferranti et al. require at least three of the five metabolic abnormalities, and central obesity is not mandatory as in the IDF criteria (Table 1).

The primary objective of this study is to propose a statistical model capable of predicting the probability of an adolescent developing MetS. The response variable of interest *y* was the presence of MetS, which takes the value “*y* = 1” if the adolescent has MetS and “*y* = 0” otherwise.

Different feed-forward ANNs were used to build the predictive model. ANNs can be applied to regression, classification, and data summarization problems, as well as in situations where there are nonlinear interactions between the dependent and independent variables. In terms of topology, to implement a neural network, we must determine the following variables: (a) the number of nodes in the input layer, (b) the number of hidden layers and the number of neurons to be inserted in these layers, and (c) the number of neurons in the output layer. The number of nodes in the input layer corresponds to the number of variables that will be used to feed the neural network. These are often the most relevant variables for the problem under study [17].

Various methods for the supervised training of ANNs have been proposed. However, the most popular algorithm used for this type of training is the backpropagation algorithm [18]. The application of the backpropagation algorithm requires the choice of a set of parameters (number of iterations of the algorithm, stopping criteria, initial weights, and learning rate), the influence of which can be decisive for the network’s ability to generalize.

The ANNs considered in this study considered the following input variables: age, sex, BMI, weight, and height. The output variable was the presence or absence of MetS (yes/no). The ANNs implemented in this study are as follows:

- ANN 1: Glycemia, WC, HDL, Triglycerides, SBP, DBP.
- ANN 2: Age, Sex, Weight, Height, SBP, DBP.
- ANN 3: Age, Sex, WC, SBP, DBP.
- ANN 4: Age, Sex, BMI, SBP, DBP.
- ANN 5: Age, Sex, WC, Weight, SBP, DBP.

ANN 1 included the same components as the criteria used in this study and was considered only as a reference network. Once the input variables were defined, the next step was to define the training and test sets. Given that three different criteria were considered for diagnosing MetS, three samples were constructed based on the prevalence of MetS according to the criterion adopted. Therefore, each sample included an equal number of adolescents with and without MetS (1:1). This sampling procedure was adopted because the criteria yielded disparate MetS prevalence rates. Adolescents without MetS were randomly selected. Each sample was divided into two subsamples: 70% for training and 30% for testing (Table 2).

To avoid overfitting, k-fold cross-validation was applied to the training set. The objective of this technique is to randomly divide the sample under study into k subsets, where each subset serves as a training set, and the remaining subsets serve as validation sets for the network. In this study, the training set was divided into ten subsets. Nine of these were used to train the network, and one subset was employed to evaluate the network’s performance. The validation process was repeated ten times. Each subset was used as both training and testing sets.

Different feed-forward networks containing only one hidden layer were implemented. The distinguishing factor was the number of neurons in the input and hidden layers. To train the network, a backpropagation algorithm was employed with a maximum of 10,000 iterations, a learning rate of 0.25, and three distinct decay parameters (0.01, 0.1, and 0.5) to prevent overfitting. The activation function used in the hidden and output layers is a sigmoidal logistic function.

To avoid overfitting, k-fold cross-validation was applied to the training set. The objective of this technique is to randomly divide the sample under study into k subsets, where each subset serves as a training set, and the remaining subsets serve as validation sets for the network. In this study, the training set was divided into ten subsets. Nine of these were used to train the network.

The generalization capacity of the trained ANN was evaluated using a test set with the following metrics: accuracy (ACUR), sensitivity (SENS), specificity (SPEC), and receiver operating characteristic (ROC) curve. These metrics were derived from a confusion matrix (Table 3) that calculated the following measures: True Positives (TP), True Negatives (TN), False Positives (FP), and False Negatives (FN).

The SENS was defined as the percentage of adolescents with MetS correctly classified by the ANN. It was calculated using SENS = TP/(TP + FN). SPEC is the percentage of adolescents without MetS correctly classified. SPEC was calculated as follows: SPEC = TN/(TN + FP). The ACUR was defined as the percentage of adolescents with and without MetS correctly classified. This was calculated using ACUR = (TP + TN)/(TP + TN + FP + FN).

A ROC curve was constructed, and the Area Under the Curve (AUC) was determined to assess the discriminatory power of the ANN. The ROC curve illustrates how the SENS and SPEC values of the method vary when different cut-off points are considered. The AUC ranges from 0 to 1. The higher the AUC, the more effective the evaluated method.

Analyses were performed in the open-access statistical program R (R Core Team, 2023). The *NeuralNetTools*, *caret*, *ggplot2*, and *pROC* packages were used (R version 4.4.0).

The data analyzed in this study are part of a larger project, Determinants throughout the Life Cycle of Obesity, Precursors of Chronic Diseases, Human Capital and Mental Health: A Contribution of the São Luís Birth Cohorts to the Unified Health System (SUS), which was approved on 29 October 2015 by the Research Ethics Committee of the University Hospital of the Federal University of Maranhão (Process n° 1.302.489).

All the participants signed an Informed Consent Form. This study was conducted in accordance with the Declaration of Helsinki and Resolution n° 466/2012 requirements of the Conselho Nacional de Saúde (National Health Council) and its complementary provisions.

## 3. Results

The study sample comprised 2064 adolescents aged 18–19, 50.9% of whom were female. Table 4 presents the descriptive measures of the study variables.

The prevalence of MetS varies depending on the diagnostic criteria used. According to the IDF and Cook et al. criteria, the prevalence rates were very similar and less than half of those obtained from the definition adopted by De Ferranti et al. (6.0%, 5.7%, and 12.3%, respectively). Regardless of the criteria chosen, lipid profile inadequacy was the most common: low HDL cholesterol levels (44.5%, 35.2%, and 22.2% in De Ferranti et al., IDF, and Cook et al., respectively) and hypertriglyceridemia (29.7% in De Ferranti et al. and 22.9% in Cook et al.), except IDF (8.4%). The IDF showed a higher prevalence of abnormal blood glucose levels (19.0%) than the other criteria (9.0% for both). Altered blood pressure showed slight variation between the criteria (9.4% to 12.6%), whereas abnormal WC was more prevalent according to the IDF (28.1%) and De Ferranti et al. (25.1%) criteria than according to the Cook et al. criteria (10.1%) (Table 5).

Table 6 presents the ANN performance for the test and total samples, as evaluated using the three criteria employed in this study. Overall, all ANNs demonstrated satisfactory performance in predicting MetS in the adolescent sample, with the ACUR, SENS, and ESPEC values exceeding 70%.

When the criteria of Cook et al. were adopted, the ANNs performed better. Among them, ANN 3 and 5 demonstrated the best performance. They used age, sex, WC, SBP, and DBP as input variables, and ANN 5 also used weight. According to the criteria of the IDF and De Ferranti, ANN 3 and ANN 4 performed better than the others.

Considering the total sample size (*n* = 2064), ANN performance slightly declined when the criteria of Cook et al. were used. However, ANN 3 and ANN 5 still demonstrated superior performance compared to the others. According to the criteria IDF, ANN 3 and ANN 5 also performed better than the others. When the criteria of De Ferranti et al. were used, ANN 3 exhibited superior performance, outperforming ANN 4, which performed better on the testing set.

**Figure 1:**
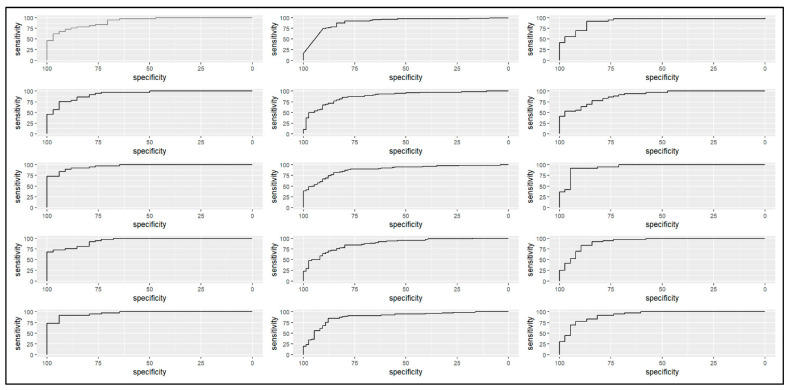
The discriminatory power of Artificial Neural Networks in predicting Metabolic Syndrome, according to the IDF, Cook et al., and De Ferranti et al. criteria, in adolescents aged 18–19. From left to right: Cook et al. (2003) [14]; De Ferranti et al. (2004) [15]; and IDF (2007) [16]. From top to bottom: ANN1; ANN2; ANN3; ANN4; and ANN5, São Luís-Maranhão, Brazil.

Figure 1 shows the ROC curves generated to assess the discriminatory power of the ANNs. The criteria of Cook et al. exhibited the highest AUC value, exceeding 90%. Additionally, ANN 5 demonstrated robust discriminatory power across all diagnostic criteria: Cook et al. (AUC = 96.7%), De Ferranti et al. (AUC = 89.0%), and IDF (AUC = 87.5%).

## 4. Discussion

This study proposed some ANNs to predict the chance of developing MetS in adolescents. Different criteria have been considered for the diagnosis of MetS. The ANN implemented using the criteria of Cook et al. demonstrated superior ACUR and SENS. Furthermore, the ANN that included only age, sex, WC, weight, SBP, and DBP (ANN 5) demonstrated superior performance and discriminatory power in predicting MetS, regardless of the criteria employed. This was followed by a network that included age, sex, WC, SBP, and DBP (ANN 3). These ANNs include the same non-invasive components used to define MetS (WC, SBP, and DBP). Moreover, the ANN used numerical values, thereby obviating the necessity of defining a cut-off point for identifying the risk of developing MetS.

WC is a practical and cost-effective anthropometric method like BMI. Nevertheless, WC exhibits an important limitation, as the diagnostic cut-off points vary according to sex and ethnic group [19]. Considering this, the criteria employed in this study considered different cut-off points for WC according to sex and age. A comparison of the SENS between ANN 3 and 5 according to the adopted criteria revealed that for ANN 3, the IDF criteria for diagnosing MetS exhibited a higher SENS for the sample under study, and for ANN 5, it was the criteria of Cook et al. This suggests that the WC cut-off point can influence identifying adolescents with MetS.

Although WC is the most used anthropometric indicator for assessing abdominal fat associated with metabolic abnormalities [7,20], it may not be a significant and predominant metabolic abnormality for defining MetS in younger people. Therefore, including other anthropometric measurements is relevant for diagnosing MetS in this age group. ANN 5’s good performance shows that incorporating weight measurement in addition to WC enables a more precise identification of MetS in adolescents when the criteria of the IDF are used.

The criteria adopted in this study use the same metabolic abnormalities to define MetS. However, their cut-off points and the necessity of WC differ in such a way as to determine significant variations [1,21]. Weihe and Weihrauch-Blüher [6] argued that it is imperative to have a unified criterion for defining MetS in children and adolescents that considers pubertal stage, ethnicity, age, and sex.

In this study, the proposed ANN for predicting MetS in adolescents aged 18–19 demonstrated satisfactory performance. To date, no studies have been identified in the literature that propose developing and validating a predictive model that considers only non-invasive measurements (WC, SBP, and DBP) in this population.

Some studies have successfully applied artificial intelligence at the primary healthcare level to track future conditions and enable early prevention to improve outcomes and optimize resources [12,22,23]. These studies demonstrated that artificial intelligence can be used to identify and predict future cardiovascular risks, allowing for implementation of early prevention strategies. This approach can potentially enhance the effectiveness of primary healthcare services and improve the population’s overall health.

In primary healthcare, an important challenge is limited access to the biochemical tests necessary for diagnosing MetS. In Brazil, access to these tests is limited, expensive, and time-consuming [24]. This can hinder the ability of primary healthcare providers to diagnose and manage MetS effectively.

Furthermore, in clinical practice, a significant number of patients either do not return for follow-up appointments or do not provide laboratory test results in a timely manner, which can delay the diagnosis of MetS. Developing an ANN to predict the chance of a patient exhibiting MetS at the initial appointment would optimize the intervention flow and contribute to managing public resources (financial, human, and material), prioritizing and guiding patients who require biochemical tests to confirm the diagnosis of MetS.

Thus, ANN 5 allows predicting the chance of an adolescent exhibiting MetS using simple and non-invasive measurements obtained during screening at a primary healthcare center with accessible and inexpensive instruments. After that, it is possible to direct efforts towards early intervention to reduce cardiometabolic risks and disease burden. The healthcare system can better manage their care by identifying adolescents who are more likely to develop MetS. This will facilitate effective interventions, including undergoing the necessary tests, attending scheduled appointments, motivation to embrace the proposed changes, and follow-up medical care. This approach is likely to result in higher intervention success rates.

The construction of an ANN for predicting MetS only considered adolescents aged 18–19. This may be a limitation because other age groups were not included in this life stage. However, the diagnostic criteria employed for MetS are limited to those applicable to this specified age range. Therefore, caution should be exercised when extrapolating the findings of this study to adolescents of various ages.

In contrast, this study used three diagnostic criteria for MetS to verify which criteria exhibited the most efficacious performance in predicting MetS. Most studies have utilized only a single criterion [4,10,20,21,25,26]. It was also determined that only input variables representing non-invasive measures that were fast and easy to obtain should be employed to serve as screening tools for MetS at the primary healthcare level. There is one study that included only complex biochemical and cardiac parameters [25].

## 5. Conclusions

The findings of this study demonstrate that ANN 3, based on age, sex, SBP, DBP, and WC, and ANN 5, with the inclusion of weight, presented better ACUR and SENS, showing great potential for screening MetS in adolescents. Therefore, non-invasive measures enable predicting the chance of an adolescent developing MetS, thereby guiding the flow of care in primary healthcare and optimizing the management of public resources.

## Figures and Tables

**Table 1:** Criteria for defining Metabolic Syndrome.

Components/ Criteria	Cook et al. (2003) [14]	De Ferranti et al. (2004) [15]	IDF (2007) [16]
SBP/DBP	≥p90	>p90 for age and sex	≥130/85 mmHg
Glycemia	≥110 mmol/L	≥110 mmol/L	≥100 mmol/L
HDL	≤40 mmol/L	<45 mmol/L (♂ 15–19 y) <50 mmol/L (♀ and ♂ < 15 y)	<40 mmol/L (♂) <50 mmol/L (♀)
Triglycerides	≥110 mmol/L	≥100 mmol/L	≥150 mmol/L
WC	≥p90 for age and sex	>p75 for age and sex	≥94 cm (♂) * ≥80 cm (♀) *

* Mandatory component for the criteria of IDF, associated with two other components. SBP: Systolic blood pressure; DBP: Diastolic blood pressure; WC: Waist circumference; p75: 75th percentile; p90: 90th percentile.

**Table 2:** Training and testing sets of Artificial Neural Networks, according to the IDF, Cook et al., and De Ferranti et al. criteria, in adolescents aged 18–19 from São Luís-Maranhão, Brazil.

Criteria	Total		Training		Test	
	MetS	No MetS	MetS	No MetS	MetS	No MetS
Cook et al.	118 (50%)	118 (50%)	81 (49.1%)	84 (50.9%)	37 (52.1%)	34 (47.9%)
De Ferranti et al.	253 (50%)	253 (50%)	175 (49.4%)	179 (50.6%)	78 (51.3%)	74 (48.7%)
IDF	124 (50%)	124 (50%)	88 (50.6%)	86 (49.4%)	36 (48.6%)	38 (51.4%)

IDF: International Diabetes Federation; MetS: Metabolic Syndrome.

**Table 3:** Confusion matrix.

Real	Prediction		Total
	MetS	No MetS	
MetS	TP	FN	TP + FN
No MetS	FP	TN	FP + TN
Total	TP + FP	FN + TN	TP + FP + FN + TN

MetS: Metabolic Syndrome; TP: True Positive; TN: True Negative; FP: False Positive; FN: False Negative.

**Table 4:** Anthropometric and clinical characteristics of adolescents aged 18–19 from São Luís-Maranhão, Brazil (*n* = 2064).

Variables	Mean ± SD (♂)|(♀)	Median (p25–p75) (♂)|(♀)	p90 (♂)|(♀)
Weight (kg)	61.3 ± 13.1 66.1 ± 12.9|56.7 ± 11.5	59.5 (160.0–174.0) 63.7 (57.4–72.4)|54.8 (48.4–63.4)	77.4 81.5|71.5
Height (cm)	167.0 ± 9.1 17.5 ± 6.9|160.7 ± 6.1	166.0 (160.0–174.0) 173.0 (169.0–178.0)|160.5 (156.5–165.0)	179.0 182.0|168.7
BMI (kg/m^2^)	21.9 ± 3.9 21.9 ± 3.7|21.9 ± 4.1	21.2 (19.1–24.0) 21.1 (19.3–23.8)|21.2 (18.9–24.1)	27.1 26.7|27.3
WC (cm)	81.7 ± 9.0 83.6 ± 9.0|79.8 ± 8.7	80.5 (75.4–86.5) 81.8 (77.3–88.2)|78.5 (73.5–84.9)	93.4 94.8|91.7
SBP (mmHg)	114.2 ± 12.2 121.0 ± 11.0|107.7 ± 9.3	113.0 (105.0–122.0) 120.0 (113.0–128.0)|107.0 (101.0–113.0)	131.0 136.0|119.0
DBP (mmHg)	70.9 ± 7.4 71.8 ± 7.6|70.0 ± 7.1	70.0 (66.0–75.0) 71.0 (66.0–76.0)|69.0 (65.0–74.0)	80.0 81.6|79.0
Glycemia (mmol/L)	92.2 ± 16.0 93.4 ± 17.8|91.1 ± 14.0	90.0 (84.0–97.0) 91.0 (85.0–98.0)|89.0 (83.0–96.0)	108.0 109.0|107.0
HDL (mmol/L)	49.4 ± 11.8 45.6 ± 10.3|53.0 ± 12.0	48.0 (41.0–56.0) 45.0 (38.0–52.0)|52.0 (45.0–60.0)	65.0 58.0|68.0
Triglycerides (mmol/L)	90.8 ± 48.5 98.3 ± 55.0|83.6 ± 40.1	79.0 (61.0–106.0) 86.0 (64.0–114.0)|73.0 (58.0–96.0)	14.7 154.0|130.0

SD: standard deviation; ♂: male; ♀: female; p25: 25th percentile; p75: 75th percentile; p90: 90th percentile; BMI: body mass index; WC: waist circumference; SBP: systolic blood pressure; DBP: diastolic blood pressure.

**Table 5:** Prevalence of Metabolic Syndrome and metabolic abnormalities, according to the IDF, Cook et al., and De Ferranti et al. criteria, in adolescents aged 18–19 from São Luís-Maranhão, Brazil (*n* = 2064).

MetS and Metabolic Abnormalities	Cook et al. (2003) [14]	De Ferranti et al. (2004) [15]	IDF (2005) [16]
MetS	5.7% (*n* = 118)	12.3% (*n* = 253)	6.0% (*n* = 124)
Waist circumference	10.1% (*n* = 208)	25.1% (*n* = 518)	28.1% (*n* = 579) *
SBP/DBP	11.5% (*n* = 238)	9.4% (*n* = 194)	12.6% (*n* = 261)
Glycemia	9.0% (*n* = 185)	9.0% (*n* = 185)	19.0% (*n* = 393)
HDL	22.2% (*n* = 459)	44.5% (*n* = 918)	35.2% (*n* = 726)
Triglycerides	22.9% (*n* = 473)	29.7% (*n* = 612)	8.4% (*n* = 174)

* Mandatory diagnostic component in IDF criteria. IDF: International Diabetes Federation; MetS: Metabolic Syndrome; SBP: systolic blood pressure; DBP: diastolic blood pressure.

**Table 6:** Artificial neural network performance for the testing set and for the total sample (*n* = 2064), according to the IDF, Cook et al., and De Ferranti et al. criteria, in adolescents aged 18–19 from São Luís-Maranhão, Brazil.

ANN	Metabolic Syndrome Criteria								
	Cook et al. (2003) [14]			De Ferranti et al. (2004) [15]			IDF (2007) [16]		
	ACUR	SENS	SPEC	ACUR	SENS	SPEC	ACUR	SENS	SPEC
*Test set*		*n* = 84			*n* = 166			*n* = 73	
ANN 1	87.3%	97.3%	76.5%	88.2%	91.0%	85.1%	85.1%	88.9%	81.9%
ANN 2	87.3%	91.9%	82.4%	82.2%	80.8%	83.8%	85.1%	88.9%	81.6%
ANN 3	88.7%	89.2%	88.2%	83.6%	80.8%	86.5%	87.8%	91.7%	84.2%
ANN 4	84.5%	89.2%	79.4%	80.3%	79.5%	81.1%	87.8%	91.7%	84.2%
ANN 5	90.1%	91.9%	88.2%	82.2%	75.6%	89.2%	80.1%	87.9%	79.6%
*Total sample*									
ANN 1	87.5%	96.6%	86.9%	87.2%	88.9%	86.9%	76.1%	93.5%	75.0%
ANN 2	84.3%	89.0%	84.0%	81.4%	77.9%	81.9%	72.8%	90.3%	71.7%
ANN 3	87.0%	89.0%	86.9%	83.8%	80.2%	84.3%	81.4%	90.3%	80.9%
ANN 4	82.4%	90.7%	81.9%	80.9%	78.7%	81.2%	77.3%	88.7%	80.9%
ANN 5	86.8%	89.8%	86.6%	85.3%	76.3%	86.5%	80.1%	87.9%	79.63%

ANN: artificial neural network; IDF: International Diabetes Federation; ACUR: accuracy; SENS: sensitivity; SPEC: specificity.

## Data Availability

Restrictions apply to the availability of these data. Data were obtained from RPS Cohort Consortium and are available from RPS Cohort Consortium with the permission of RPS Cohort Consortium.

## References

[1] Melo D.A., Santos A.M., Silveira V.N.C., Silva M.B., Diniz A.S. (2023). Prevalence of metabolic syndrome in adolescents based on three diagnostic definitions: A cross-sectional study. Arch. Endocrinol. Metab..

[2] DeBoer M.D. (2019). Assessing and Managing the Metabolic Syndrome in Children and Adolescents. Nutrients.

[3] Al-Hamad D., Raman V. (2017). Metabolic syndrome in children and adolescents. Transl. Pediatr..

[4] Al-Shami I., Alkhalidy H., Alnaser K., Mukattash T.L., Al Hourani H., Alzboun T., Orabi A., Liu D. (2022). Assessing metabolic syndrome prediction quality using seven anthropometric indices among Jordanian adults: A cross-sectional study. Sci. Rep..

[5] Orsini F., D’Ambrosio F., Scardigno A., Ricciardi R., Calabrò G.E. (2023). Epidemiological Impact of Metabolic Syndrome in Overweight and Obese European Children and Adolescents: A Systematic Literature Review. Nutrients.

[6] Weihe P., Weihrauch-Blüher S. (2019). Metabolic Syndrome in Children and Adolescents: Diagnostic Criteria, Therapeutic Options and Perspectives. Curr. Obes. Rep..

[7] Nagayama D., Sugiura T., Choi S.Y., Shirai K. (2022). Various Obesity Indices and Arterial Function Evaluated with CAVI—Is Waist Circumference Adequate to Define Metabolic Syndrome?. Vasc. Health Risk Manag..

[8] do Vale Moreira N.C., Hussain A., Bhowmik B., Mdala I., Siddiquee T., Fernandes V.O., Júnior R.M.M., Meyer H.E. (2020). Prevalence of Metabolic Syndrome by Different Definitions, and Its Association with Type 2 Diabetes, Pre-Diabetes, and Cardiovascular Disease Risk in Brazil. Diabetes Metab. Syndr..

[9] Vanlancker T., Schaubroeck E., Vyncke K., Cadenas-Sanchez C., Breidenassel C., González-Gross M., Gottrand F., Moreno L.A., Beghin L., Molnár D. (2017). Comparison of Definitions for the Metabolic Syndrome in Adolescents. The HELENA Study. Eur. J. Pediatr..

[10] El-Wahab E.W., Shatat H.Z., Charl F. (2019). Adapting a Prediction Rule for Metabolic Syndrome Risk Assessment Suitable for Developing Countries. J. Prim. Care Community Health.

[11] Hirose H., Takayama T., Hozawa S., Hibi T., Saito I. (2011). Prediction of metabolic syndrome using artificial neural network system based on clinical data including insulin resistance index and serum adiponectin. Comput. Biol. Med..

[12] Wang F.-H., Lin C.-M. (2020). The Utility of Artificial Neural Networks for the Non-Invasive Prediction of Metabolic Syndrome Based on Personal Characteristics. Int. J. Environ. Res. Public Health.

[13] Confortin S.C., Ribeiro M.R.C., Barros A.J., Menezes A.M.B., Horta B.L., Victora C.G., Barros F.C., Gonçalves H., Bettiol H., Santos I.S.D. (2021). RPS Brazilian Birth Cohorts Consortium (Ribeirão Preto, Pelotas and São Luís): History, objectives and methods. Cad. Saúde Pública.

[14] Cook S., Weitzman M., Auinger P., Nguyen M., Dietz W.H. (2003). Prevalence of a Metabolic Syndrome Phenotype in Adolescents: Findings from the third National Health and Nutrition Examination Survey, 1988–1994. Arch. Pediatr. Adolesc. Med..

[15] De Ferranti S., Gauvreau K., Ludwig D.S., Neufeld E.J., Newburger J.W., Rifai N. (2004). Prevalence of the Metabolic Syndrome in American Adolescents: Findings from the Third National Health and Nutrition Examination Survey. Circulation.

[16] Zimmet P., Alberti K.G.M.M., Kaufman F., Tajima N., Silink M., Arslanian S., Wong G., Bennett P., Shaw J., Caprio S. (2007). The metabolic syndrome in children and adolescents—An IDF consensus report. Pediatr. Diabetes.

[17] Santos A.M., Seixas J.M.D., Pereira B.D.B., Medronho R.D.A. (2005). Usando redes neurais artificiais e regressão logística na predição da hepatite A. Rev. Bras. Epidemiol..

[18] Haykin S. (2009). Neural Networks and Learning Machines.

[19] Fang H., Berg E., Cheng X., Shen W. (2018). How to best assess abdominal obesity. Curr. Opin. Clin. Nutr. Metab. Care.

[20] Kwon E., Nah E.-H., Kim S., Cho S. (2021). Relative Lean Body Mass and Waist Circumference for the Identification of Metabolic Syndrome in the Korean General Population. Int. J. Environ. Res. Public Health.

[21] Lee S., Lee H., Choi J.R., Koh S.B. (2020). Development and Validation of prediction Model for Risk Reduction of Metabolic Syndrome by Body Weight control: A prospective population-based Study. Sci. Rep..

[22] Gallardo-Rincón H., Ríos-Blancas M.J., Ortega-Montiel J., Montoya A., Martinez-Juarez L.A., Lomelín-Gascón J., Saucedo-Martínez R., Mújica-Rosales R., Galicia-Hernández V., Morales-Juárez L. (2023). MIDO GDM: An innovative artificial intelligence-based prediction model for the development of gestational diabetes in Mexican women. Sci. Rep..

[23] Wang J., Lv B., Chen X., Pan Y., Chen K., Zhang Y., Li Q., Wei L., Liu Y. (2021). An early model to predict the risk of gestational diabetes mellitus in the absence of blood examination indexes: Application in primary health care centers. BMC Pregnancy Childbirth.

[24] Do Nascimento L.C., Viegas S.M.F., Menezes C., Roquini G.R., Santos T.R. (2020). The SUS in the lives of Brazilians: Care, accessibility and equity in the daily lives of Primary Health Care users. Physis.

[25] Mao L., He J., Gao X., Guo H., Wang K., Zhang X., Yang W., Zhang J., Li S., Hu Y. (2018). Metabolic syndrome in Xinjiang Kazakhs and construction of a risk prediction model for cardiovascular disease risk. PLoS ONE.

[26] Motamed N., Ajdarkosh H., Karbalaie Niya M.H., Panahi M., Farahani B., Rezaie N., Nikkhah M., Faraji A.H., Hemmasi G., Perumal D. (2022). Scoring systems of metabolic syndrome and prediction of cardiovascular events: A population based cohort study. Clin. Cardiol..

